# Carob pulp flour as an innovative source of bioactive molecules for the preparation of high-value-added jellies

**DOI:** 10.1016/j.heliyon.2024.e38354

**Published:** 2024-09-25

**Authors:** Umile Gianfranco Spizzirri, Luigi Esposito, Paolino Caputo, Maria Martuscelli, Martina Gaglianò, Maria Lisa Clodoveo, Giuseppina De Luca, Cesare Oliverio Rossi, Marco Savastano, Eva Scarcelli, Monica Rosa Loizzo, Donatella Restuccia, Francesca Aiello

**Affiliations:** a Ionian Department of Law, Economics and Environment, University of Bari Aldo Moro, 74123, Taranto, Italy; b Department of Bioscience and Technology for Food, Agriculture and Environment, University of Teramo, 64100, Teramo, Italy; c Department of Chemistry and Chemical Technologies & UdR INSTM, University of Calabria, 87036, Rende, Italy; d Interdisciplinary Department of Medicine, University of Bari Aldo Moro, 70125, Bari, Italy; e Department of Management, Sapienza University of Rome, Via Del Castro Laurenziano 9, 00161, Rome, Italy; f Department of Pharmacy, Health and Nutritional Sciences, University of Calabria, 87036, Rende, Italy

**Keywords:** Carob pulp flour, Jelly, Active molecules, Antioxidant, Rheological, Sensorial, Hypoglycemic

## Abstract

The rising demand for healthier alternatives to traditional sugary products has driven the exploration of natural substitutes for sucrose. This study aimed to investigate carob pulp flour (CPF) as a viable alternative to sucrose in the production of high-value jellies with enhanced health benefits. Carob pulp flour was spectroscopically characterized, revealing the presence of bioactive molecules, such as natural antidiabetic polyols and polyphenols. Colorimetric tests demonstrated a significant concentration of polyphenolic molecules in CPF, with a remarkable scavenging activity against radical species in both organic and aqueous environments. Jellies based on CPF (CGC) were successfully prepared and exhibited strong antioxidant activity against ABTS (IC_50_ 0.158 mg mL^−1^) and DPPH (IC_50_ 0.175 mg mL^−1^) radicals, maintaining their properties over 15 days, unlike the sucrose-based control, which showed no antioxidant activity. The CPF-enhanced jellies consistently demonstrated higher G′ values (in the range 20–35 °C) than the sucrose-based jellies, indicating improved consistency, elasticity, and strong gel properties, even at higher temperatures. Sensory analysis revealed significant differences, with CPF-based jellies displaying enhanced chocolate (2.3 ± 1.0), ripe fruit (2.3 ± 1.8), and caramel odors (2.0 ± 0.9), as well as increased bitterness and astringency, reduced sweetness, and improved texture. Additionally, CPF-based jellies exhibited significant hypoglycemic properties, with dose-dependent inhibitory effects on α-amylase (57.7 %) and α-glucosidase (50.3 %), and a moderate lipase inhibitory effect (48.6 %) at the maximum concentrations tested. The findings of this study highlight the potential of CPF as a functional ingredient in the food industry, offering a healthier alternative to sucrose in jelly production. The inclusion of CPF not only enhances the antioxidant and sensory properties of jellies but also contributes to significant hypoglycemic effects, making it a promising candidate for the development of functional foods with added health benefits.

## Highlights

• Carob pulp flour contains strong antioxidant bioactive compounds.
• Carob-based jellies display enhanced rheological properties even at higher temperatures.
• Carob-based jellies exhibit chocolate, ripened fruit, and caramel odors.
• The jellies show significant hypoglycemic effects.
• Carob-based jellies are a healthier alternative to sucrose-based jellies.

## 1 Introduction

Recently, lesser known fruits, such as carob, have gained popularity. These fruits are rich in both non-nutritive and nutritive compounds, including phenolic and flavonoid molecules. They offer unique flavors and tastes, as well as outstanding medicinal properties and health benefits [1]. Carob (*Ceratonia siliqua* L.) is an evergreen tree that is part of the *Leguminosae* family widespread throughout the Mediterranean region and highly valued for its fruits [2]. According to the Food and Agriculture Organization's 2022 data, Portugal is the world's leading producer and carob tree cultivation. Italy is the second-largest producer, with significant production also in Morocco, Turkey, and Greece [3]. The economic significance of the carob tree in the industrial field is related to the employment of carob gum derived from the carob fruit [4]. Carob pulp, a by-product traditionally used for animal feed, can be milled into carob flour for human consumption. Rich in nutrients and secondary metabolites, carob pulp offers health benefits including antioxidant, anti-inflammatory, and anti-aging effects, as well as in the treatment of the metabolic syndrome (MetS).

Metabolic Syndrome (MetS) is a multifaceted condition frequently linked to insulin resistance and elevated cholesterol levels [5]. Although research has shed light on many of the mechanisms involved in MetS, its treatment remains a significant challenge. As a result, numerous research teams are exploring biological molecules in foods that could help prevent this disorder. Many of these compounds, particularly phenols, exhibit both anti-hypercholesterolemic and antihyperglycemic properties [6]. One of the most widely used strategies to mitigate MetS and obesity involves inhibiting enzymes like α-glucosidase, α-amylase, and lipase. Inhibiting pancreatic lipase, for instance, decreases fat absorption, leading to a hypolipidemic effect, while blocking carbohydrate-hydrolyzing enzymes slows down carbohydrate digestion, resulting in an antihyperglycemic outcome [7,8].

The presence of high-value biological compounds in the carob pulp makes this raw material an attractive plant source with notable biological activity [[9], [10], [11]]. In particular, carob pulp flour (CPF) represents a source of antioxidant molecules as well as a valuable substitute of sucrose in the preparation of food products with significant health benefits. Literature data examine the potential of natural sweeteners as alternatives to sucrose, highlighting their health benefits and challenges in food applications [12]. Sweeteners like stevia, erythritol, xylitol, and monk fruit are praised for their ability to reduce sugar intake without compromising taste, offering properties such as antidiabetic effects, antioxidant activity, and low calories [13]. The impact of replacing sucrose with options like honey, agave nectar, and coconut sugar is also explored, showing benefits like a lower glycemic index and additional nutrients, though they come with considerations such as caloric content and possible allergies [14]. Non-caloric sweeteners like steviol glycosides and mogrosides are noted for their effectiveness in managing weight and blood sugar levels, making them suitable for diet-conscious consumers [15]. In the development of low-calorie confectionery, the importance of careful formulation to preserve taste and texture has been emphasized, and the role of the natural sweeteners and the inconvenient in altering the sensory experience [16]. Overall, while these alternatives are valuable in reducing sugar and enhancing health, the challenge remains in replicating the sensory qualities of sucrose in food products.

In this study, carob pulp flour has been successfully used as a substitute for sucrose in jelly production. This substitution not only offers considerable health benefits but also preserves desirable sensory characteristics. The results demonstrate that carob pulp flour is a viable alternative for creating healthier jellies without compromising taste and texture. Chemical and biological characterization of carob pulp flour, carried out using spectroscopic and colorimetric techniques, enabled the identification of the main molecules present in the plant-based derivatives. Sucrose-free CPF-based jellies were successfully prepared and thoroughly characterized in terms of antioxidant, rheological, and sensory properties as function of the time. Finally, to emphasize the health benefits of the proposed formulation, *in vitro* tests were performed to evaluate its hypoglycaemic effects.

## 2 Materials and methods

### 2.1 Chemicals and reagents

All the reagents were purchased from Phytolab (Aprilia, Noale, Italy), Merck (Darmstadt, Germany) and VWR International (Milan, Italy). HPLC-grade water was provided by Merk Life Science Srl (Milan, Italy) and were of analytical grade or higher, unless otherwise specified.

### 2.2 Carob pulp flour

Carob pulp flour (CPF) (Amele cv) was supplied by Masseria Agricola Olère (Contrada San Salvatore, 10, 40017 Ostuni, Brindisi, Italy). A summary of its main nutritional values is presented in Table 1.

**Table 1:** Nutritional information of the organic carob pulp flour as reported on the label.

Average Values for 100 g of Product		% RDA
Energy value (Kcal)	371	
Fat (g)	0.30	0.40
of which is saturated (g)	<0.10	<0.10
Carbohydrate (g)	41.30	15.48
of which is sugar (g)	36.50	
Fiber (g)	6.50	26.00
Protein (g)	4.70	9.40
Salt (mg)	0.00	0.00
Potassium (mg)	800.00	39.00
Vitamin B_2_ (mg)	0.45	32.00

#### 2.2.1 NMR sample preparation and experiments

The chemical composition of the initial CPF was conducted by NMR spectroscopy. A sample was prepared using 30 mg of CPF solubilized in 550 μL of D_2_O and 20 μL of a solution of 0.16 % (w/w) sodium azide (NaN_3_) and 0.11 % (w/w) of 3-(trimethylsilyl)propionic-2,2,3,3-d_4_ acid (TMSP-d_4_) in D_2_O. Deuterated water was used for locking the signal, TMSP-d_4_ was used for referencing chemical shift, and NaN_3_ was used to prevent the onset of bacteria during the recording of multinuclear NMR experiments. The whole mixture was transferred into a 5 mm NMR tube which was closed and inserted into the NMR spectrometer. NMR measurements were performed on a Bruker Avance spectrometer operating at 500 MHz for 1H (11.74 T), using a 5 mm multinuclear probe TBO (triple-resonance broadband observe) and a standard variable-temperature control unit BVT-3000 (Bruker, Fällanden, Switzerland). The sample temperature was established at 298 K. Spectral assignments were performed on the basis of the one-dimensional ^1^H, ^13^C-{^1^H} NMR spectra, bi-dimensional homo and heteronuclear correlation NMR experiments (^1^H–^1^H COSY, ^1^H-^13^C HMQC) and by comparison with published data [17]. ^1^H NMR spectrum was recorded using the noesypr1d Bruker standard sequence (presaturation for suppression of the residual water signal), a SW of 14.00 ppm, a D1 of 4.0 s and collecting 256 FIDs points. ^13^C NMR spectrum was recorded with an inverse gated-decoupling pulse sequence (zgig Bruker pulse sequence), with a spectral width of 250 ppm, 64 K data points, a D1 of 5 s, an AQ of 1.04 s and by collecting 6000 scans. The COSY experiment (cosygpprqf Bruker pulse program) was acquired using a SW of 14.00 ppm on both dimensions, 2K data points, 28 scans and 256 increments, the ^1^H-^13^C HMQC spectrum (hmqcgpqf Bruker pulse program) was recorded using a SW of 14.00 ppm (^1^H) and 250.00 ppm (^13^C), 2K datapoints, 512 scans and 32 experiments.

### 2.3 Jellies preparation

Carob pulp flour was used in the preparation of jellies (CGC), which were analyzed over time to evaluate antioxidant activity. For the production of 20 jellies, each weighing approximately 1.2–1.5 g, the following ingredients were used: commercial gelatin (5.0 g), CPF (8.5 g), and 35 mL of H_2_O. The procedure was adapted from literature protocols with modifications [18]. The commercial gelatin was dissolved in 35 mL of H_2_O at 50 °C under stirring. After complete dissolution of the gelatin, CPF was added, and once all ingredients were fully integrated, the warm semi-liquid mixture was poured into molds. The molds were refrigerated at 4 °C overnight to solidify. The ratios of gelatin to water and gelatin to CPF were carefully selected to optimize the rheological, mechanical, and bioactive properties of the jellies. Similarly, control jellies (CGS) were prepared where sucrose completely replaced CPF.

All samples were analyzed for antioxidant activity over 15 days, starting on day 0, followed by assessments on day 7 and day 15. The analysis procedure was adapted from the literature with some modifications [19]. For extraction, 1.0 g of each sample was suspended in 10 mL of an aqueous methanol solution (80/20 v/v) with 0.1 mL of HCl (37 % w/w). The mixture has been sonicated (15 min at 20 °C) and then left to stand at 4 °C for 24 h. The sample has been sonicated again (15 min at 20 °C) and centrifuged (at 4000 rpm for 10 min). The resulting supernatant was collected and analyzed for phenolic content and antioxidant properties.

### 2.4 Antioxidant features of CPF and jellies

#### 2.4.1 Phenolic compounds quantification

The total phenolic content (TPC) of organic CPF and jellies was measured using the Folin–Ciocalteu method, following a tailored procedure adapted from previous studies [20]. An aqueous solution of CPF (1.0 mg mL^−1^, 1.0 mL) was mixed with the Folin-Ciocalteu reagent (6 mL) and Na_2_CO_3_ solution (3.0 mL, 2 % w/v). The mixture was stirred in the dark for 2 h, after which the absorbance was measured at 760 nm using a spectrophotometer (Evolution 201, Thermo Fisher Scientific, Hillsboro, OR, USA). A control sample was prepared by replacing CPF with purified water, while keeping all other reagents the same. TPC was expressed as milligrams of gallic acid (GA) per gram of dry sample (mg GA g^−1^). A calibration curve was built using GA solutions (8.0–40.0 μmol L^−1^). TPC tests on the jellies were conducted at different time points (time = 0, 7, and 15 days).

#### 2.4.2 Total phenolic acid quantification

The phenolic acid content (PAC) in CPF and jellies was determined using the Arnov test, with certain modifications [21]. In brief, 1.0 mL of an aqueous solution of CPF (1.0 mg mL^−1^) was combined with HCl (1.0 mL, 0.5 mol L^−1^), NaOH (1.0 mL, 4.0 % w/v), 1.0 mL of Arnov's reagent (a mixture of 0.1 mg mL^−1^ sodium nitrite and 0.1 mg mL^−1^ sodium molybdate), and H_2_O to bring the total volume to 10 mL. The absorbance was then measured at 490 nm using a spectrophotometer. PAC was expressed in milligrams of GA per gram of sample (mg GA g^−1^), using a calibration curve for quantification. PAC measurements for the jellies were carried out at different time intervals (time = 0, 7, and 15 days).

#### 2.4.3 Total flavonoid quantification

The flavonoid content (FC) in CPF and jellies has been evaluated spectrophotometrically using a methodology adapted from the literature apporting some slight changes [22]. In brief, 0.5 mL of a solution of CPF (1.0 mg mL^−1^) was mixed with NaNO_2_ (0.15 mL, 15 % w/v) and 2.0 mL of H_2_O. After 6 min, AlCl_3_ solution (0.15 mL, 10 % w/v) has been added, followed by NaOH solution (3.0 mL, 4 % w/v), and the volume was brought up to 5 mL using H_2_O. Six minutes later, the mixture has been left to incubate in the dark for 15 min. The absorbance was then measured at 510 nm using a spectrophotometer. The flavonoid content was expressed as milligrams of catechin (CT) per gram of sample (mg CT g^−1^). Analyses of the jellies were conducted at different time intervals (time = 0, 7, and 15 days).

#### 2.4.4 Scavenger performances

Free radical scavenger properties of CPF and jellies were assessed against ABTS (2,2′-azino-bis(3-ethylbenzothiazoline-6-sulphonic acid)) and DPPH (2,2-diphenyl-1-picrylhydrazyl) radicals. To assess the scavenging effect in an aqueous environment, 0.5 mL of an aqueous solution of the sample (0.01–0.10 mg mL^−1^) has been added to 2.0 mL of ABTS radical solution. The mixture was left to incubate for 5 min at 37 °C, followed by spectrophotometric measurement at 734 nm [23].

The scavenging activity in an organic medium has been assessed by mixing 5.0 mL of the sample solution (0.01–0.10 mg mL^−1^) with 5.0 mL of an alcoholic solution of DPPH (200 μmol L^−1^). The mixture was incubated at 25 °C for 30 min, and the lipophilic radical was spectrophotometrically quantified at 517 nm [24].

The inhibition (%) of both radical species was determined using equation (1):

(1) Inhibition (%) = (A_0_ - A_1_) / A_0_ x 100

In which A_0_ represents the absorbance of the control, while A_1_ is the absorbance of the sample. The scavenging activity of the sample is reported as IC_50_. As positive control the ascorbic acid has been employed.

### 2.5 Dynamic rheological quantifications

Rheological measurements were carried out using a shear-stress-controlled SR5000 rheometer (Rheometrics, U.S.A.) equipped with a plate-plate geometry (25 mm diameter; 1.00 mm gap). The control of the temperature control was performed through a Peltier system (accuracy equal to ±0.1 °C). The measurements involved the application of a sinusoidal stress at each jelly. The in-phase strain component relative to the applied stress was used to calculate the storage or elastic modulus (G′), whereas the out-of-phase strain component was used to define the loss or viscous modulus (G″). The applied stress remained within the viscoelastic region (100 Pa). Prior to testing, stress sweep tests at 1 Hz were conducted to determine the linear sweep-strain range. The values of G′ and G″ were measured over a frequency range equal to 0.1–60 Hz. These frequency sweep tests were performed at different temperatures (20, 25, 30, and 35 °C), at two time intervals: at the initial time (zero) and after one week.

### 2.6 Sensory analysis of jellies

Jellies were undergone to a qualitative descriptive analysis (QDA) test for assessing sensory properties and adequate descriptors for specific products. Participants were voluntarily taking part to each session, all of them filled the informed consent donating required information (sex and age) and agreed about privacy data protection, according to Generally Data Protection Regulation (GDPR regulation 2016/679). Ten panelists were recruited (6 man and 4 woman) from 26 to 35 years old (average age 29.7) among personal of the Department of Bioscience from the University of Teramo (Italy).

The QDA test followed these steps. Firstly, the abilities in distinguishing odors and tastes were screened. Multiple training sessions were done in which triangular testes of samples were served and scores were individually reported through lines scales according to guidelines from the Society of Sensory Professionals and ISO [[25], [26], [27]]. The training was considered complete once the judges demonstrated discriminative ability, consistency in their assessments, and sufficient consensus. After this, proper descriptors and a common vocabulary for scoring samples were individuated. The panel leader worked with panelists to drive the discussion without interfering or affecting any decision. The final step consisted of sampling jellies. 2 × 2 cm squared jellies were served and coded with a random numeration. Each panelist was given of individual templates where descriptors were divided for five classes: odor, taste, aftertaste, flavor, and textural properties. Before the beginning of the session panelists were asked to rate each descriptor in a scale from 1 to 5 where 1 meant absence of the attribute while 5 was the maximum value of perception. Moreover, each descriptor was read and explained again to be sure of the agreed meaning. Panelists were also asked to rinse their mouth between the two samples with still water. Judges were seated in individual locations inside the laboratories of the Department of Biosciences, Food Science and Technology of the University of Teramo, that match requisites for sensory tests as reported in in the ISO 8589:2007.

### 2.7 α-Glucosidase and α -amylase inhibitory effect

The α -glucosidase and α -amylase inhibitory properties has been assessed following the procedure previously reported previously reported [28,29]. For the evaluation of α-glucosidase inhibitory activity, a maltose solution was prepared by dissolving the maltose (12.0) g in 300 mL of a 50 mM sodium acetate buffer. Simultaneously, the dissolution of 1.0 mg of α-glucosidase (EC 3.2.1.20, activity of 10 units mg⁻^1^) in 10.0 mL of ice-cold H_2_O allowed the preparation of the enzyme solution. Additionally, solutions of O-dianisidine (DIAN) and the peroxidase/glucose oxidase (PGO) system-color reagent were prepared according to the manufacturer's instructions. The test involved the mixing of 5 μL of the extract (25 - 1000 μg mL^−1^) with 250 μL of the maltose solution and 5 μL of the α -glucosidase solution at 37 °C for 30 min. After the incubation, perchloric acid (50 μL) has been added, and the mixture was centrifuged. The resulting supernatant was then mixed with DIAN (5 μL) and PGO reagent (300 μL) and incubated again at 37 °C for 30 min. Absorbance was measured at 500 nm using a UV–Vis spectrophotometer (Jenway 6003, Carlo Erba, Milan, Italy).

The α-amylase inhibitory assay has been performed preparing a suitable starch solution (125.0 mg of potato starch in a sodium phosphate buffer (20 mM) containing sodium chloride (6.7 mmol L^−1^)). In this assay, 200 μL of the starch solution were added to 40 μL of the sample at different concentrations (25 - 1000 μg mL^−1^) and enzyme solution (prepared by dissolving 0.0253 g of α-amylase [EC 3.2.1.1] in 100 mL of cold H_2_O in 5 min at 25 °C). After incubation, the absorbance was recorded at 540 nm. In both assays, acarbose served as a positive control.

### 2.8 Pancreatic lipase inhibitory effect

To evaluate the impact of the extract on pancreatic lipase activity, a protocol using a 96-well plate was employed, based on a previously described method [30]. In summary, 25 μL of the extract, at varying concentrations (2.5–40 mg mL^−1^), were combined with 6 μL of porcine pancreatic lipase (EC 3.1.1.3) (1.0 mg mL^−1^), 4-nitrophenyl octanoate (5 mmol L^−1^) in DMSO, and a Tris-HCl buffer solution in water (pH 8.5). After incubating the mixture at 37 °C for 30 min, the absorbance was recorded at 405 nm using a UV–Vis spectrophotometer (Jenway 6003, Carlo Erba, Milan, Italy). Orlistat was employed as the positive control.

### 2.9 Statistical analysis

Antioxidant assays were performed in triplicate, with data expressed as means ± SD. Statistical analysis was conducted using the Wilcoxon test, and a p-value of less than 0.05 was considered statistically significant. All data analyses were performed using GraphPad Prism 8.3.0 (GraphPad Software, Inc., San Diego, CA, USA).

For the sensory evaluation, statistical significance (p < 0.05) was quantified employing one-way ANOVA for paired samples, conducted with Microsoft Excel for Mac (version 16.78.3).

## 3 Result and discussions

### 3.1 Characterization of the carob pulp flour

#### 3.1.1 NMR characterization

**Fig. 1:**
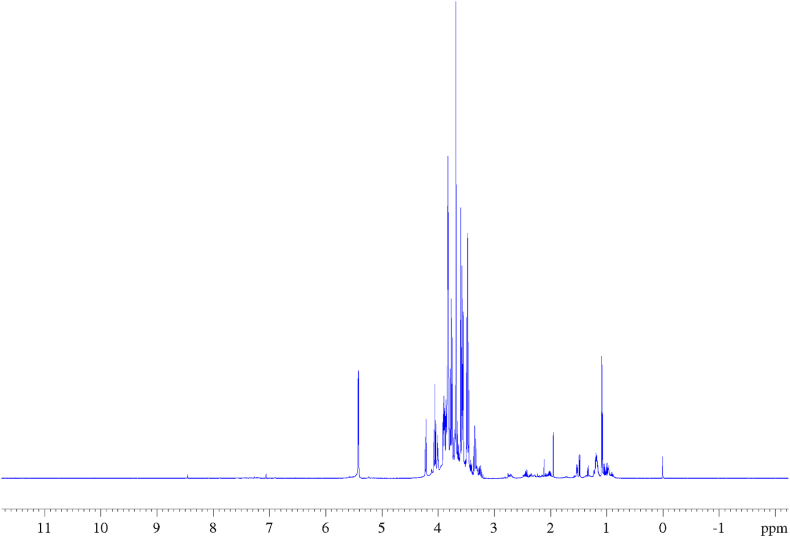
500 MHz ^1^H NMR spectrum of carob flour in D_2_O.

**Fig. 2:**
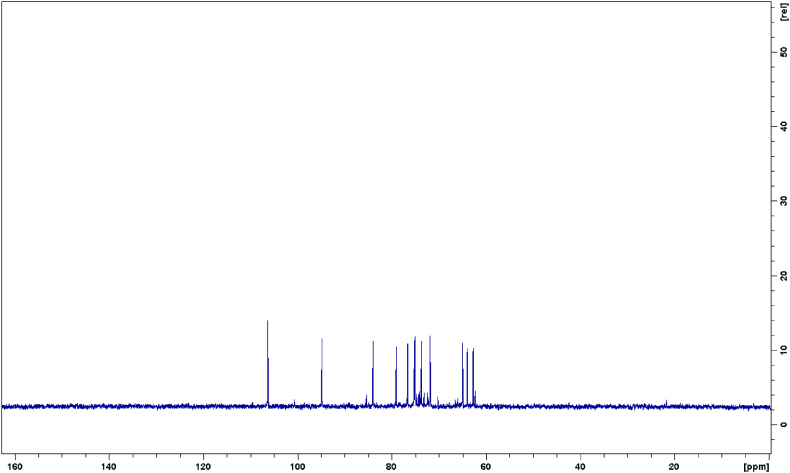
500 MHz ^13^C-{^1^H} NMR spectrum of carob flour in D_2_O.

**Fig. 3:**
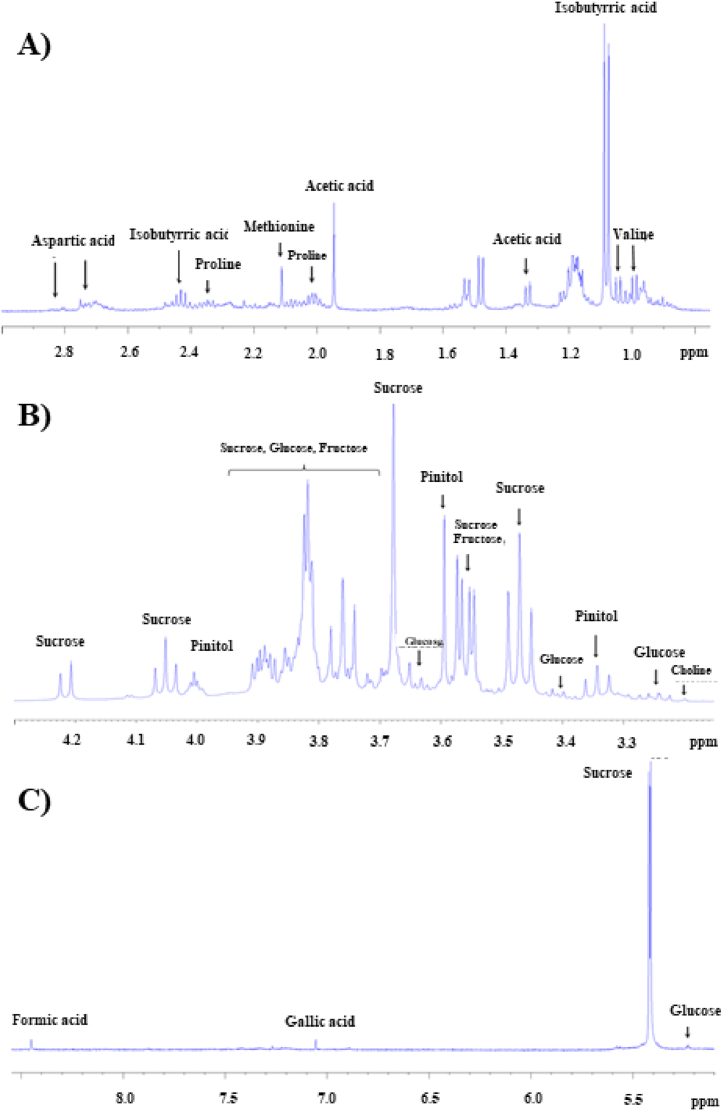
Expanded regions of ^1^H NMR spectrum of carob flour and signals assignment: **A)** from 0.74 to 3 ppm; **B)** from 3.15 to 4.3 ppm and **C)** from 5 to 8.5 ppm.

In Fig. 1, Fig. 2 the ^1^H and ^13^C NMR experiments recorded on the CPF sample have been reported. In the ^1^H NMR spectrum, three distinct regions are observed: the first region, from 0 to 3 ppm, includes the signals of aliphatic protons from organic acids and free amino acids; the second region, from 3 to 6 ppm, corresponds to sugars and sugar-like compounds; and finally, the region between 6 and 8 ppm represents the aromatic compounds. In Fig. 3(A–C) these regions are enlarged and report the attributions of all recognized metabolites. This identification process was quite laborious due to the complexity of the proton spectrum, quite common for these natural mixtures consisting of multiple compounds, which makes its interpretation and the identification of individual components very difficult. This complexity is due to several factors: a) a single molecule produces more than one signal; b) overlapping of signals belonging to different molecules which makes it difficult to determine the J-couplings and the exact nature of the signal through the one-dimensional ^1^H NMR spectrum alone; c) low intensities of some signals due to the low concentration of some metabolites. However, despite this complexity and the micromolar limit of detection (LOD), high-resolution NMR offers several advantages over other analytical techniques (e.g. LC-MS): it is non-biased, non-invasive, non-destructive, does not break analytes and permits the employment of matrices without the need for extraction, chromatographic separation or chemical derivatization of the sample. Moreover, it is fast and multinuclear allowing the simultaneous identification of a wide range of metabolites (such as sugars, organic acids, alcohols, polyols etc.) in a highly reproducible manner thanks to 1D and 2D experiments. The approach used in this work to assign each signal to the corresponding metabolites of the CPF sample involved, in addition to the use of one-dimensional ^1^H and ^13^C-{^1^H} NMR spectra, the use of 2D ^1^H–^1^H COSY and ^1^H-^13^C HMQC correlation experiments (reported in the SI in Figs. S1 and S2, respectively), the employment of data available in the literature and, crucial in the identification process, the use of the HMDB (https://hmdb.ca) database and sophisticated data processing software such as CHENOMX (https://www.chenomx.com), which include chemical shift calculations based on the pH matrix value. Most of the recognized and assigned metabolites in the examined CPF sample (Fig. 3 and Table 2) belong to organic acids, carbohydrates, amino acids, and a few other compounds such as D-pinitol and choline. The content of polyphenols in carob flour is known from the literature [10], however in the sample analyzed here their low concentration allows to identify only gallic acid, the most abundant phenolic molecule in the carob matrices [31]. Indeed, gallic acid shows a singlet at 7.05 ppm in the ^1^H NMR spectrum which is clearly correlated to the ^13^C NMR signal at 107 ppm in the HMQC experiment. In the 1D ^1^H NMR spectrum it was possible to recognize several signals corresponding to other organic acids. For example, for lactic acid the doublet signal of the CH_3_ group in beta to the carboxylic group at 1.22 ppm was identified and, following the connectivity information obtained from the 2D spectra, COSY and HMQC respectively, it was also possible to attribute the resonance of the proton in alpha to the carboxylic group at 3.71 ppm and the resonance of ^13^C (-CH_3_) at 21 ppm. Again, for isobutyric acid it is easy to recognize in the ^1^H spectrum the doublet at 1.08 ppm attributable to the two CH_3_ groups in beta to the carboxylic group (β-CH_3_) and, as before, following the connectivity of the COSY spectrum it was also possible to assign the resonance of the proton in alpha to the carboxylic group at 2.43 ppm (α-CH); while following the connectivity of the HMQC spectrum it was also possible to assign the resonance of α-CH and β-CH_3_ carbons respectively at 36.2 and 18.8 ppm. By following the same procedure and using information from the HMDB database, it was possible to recognize the signals of the other organic acids and of many amino acids as reported in Fig. 3 and Table 2. The region of the ^1^H NMR spectrum that goes from 3 to 6 ppm is the most crowded and characteristic of carbohydrates and inositols. To identify the molecules of sugar present in the sample, in addition to taking into account the few isolated signals in the ^1^H NMR spectrum, extensive use was made of the information available from the spectra of the individual sugars present in the HMDB database. Inositols are stereoisomers of hexahydroxy cyclohexane. In a work reported by Abdul in 2022 [32] six inositols and their derivatives (methyl ethers) were isolated and characterized from carob, but in the spectrum of the sample under examination only the D-pinitol signals were recognized [33]. This compound is of nutraceutical interest as it owes several health benefits against different diseases [32,34]. Many of the signals of pinitol are recognizable in the 1D proton spectrum and by following the COSY spectrum connectivity it was possible to attribute all the proton signals of the compound. Hence, the triplet at 3.34 ppm was attributed to the proton in position 3 (see Table 2 for the numbering) which is coupled, with the same J coupling value, to the proton in position 4 at 3.66 ppm and to the proton in position 2 at 3.81 ppm; the singlet at 3.59 ppm is due to the three protons of the -OCH_3_ group; and finally the multiplet at 4.00 ppm is attributed to the protons in positions 1 and 6. The ^13^C chemical shifts reported in Table 2 were assigned taking into account the 2D HMQC correlation spectrum. D-pinitol has been isolated in the past from natural matrices and studied by NMR [34]. The chemical shifts and the nature of the signals detected in the spectra of the CPF sample analyzed here fit perfectly with the data obtained for the isolated molecule. By following this comprehensive approach, it was possible, starting directly from a sample of untreated carob pulp flour, to obtain a Carob-Profiling based on NMR and to perform the resonance assignment of seventeen compounds, which are reported in Table 2 and Fig. 3 together with all the experimental information obtained from the NMR spectra.

**Table 2:** ^1^H and^13^C chemical shifts of compounds in Carob Flour.

Compound	Assignment	^1^H (ppm)	Multiplicity	^13^C (ppm)
Amino acids				
Valine	γ-CH_3_	0.99	d	
	γ′-CH_3_	1.04	d	
Alanine	α-CH	3.79	d	
	β-CH_3_	1.47		
Proline	α-CH	4.13	m m	
	β-CH	2.33		
	β′-CH	2.06		
	γ-CH_2_	1.99		
	δ-CH	3.40		
	δ′-CH	3.33		
Methionine	−CH_3_	2.11		
Carbohydrates				
α-glucose	CH-1	5.22	d	94.83
	CH-2	3.53		
	CH-3	3.70		
	CH-4	3.46		
	CH-5	3.83		
	CH_2_-6,6	3.77; 3.83		
β-glucose	CH-1	4.64	dd	98.65
	CH-2	3.23		76.93
	CH-3	3.48		78.54
	CH-4	3.38		72.38
	CH-5	3.46		78.62
	CH_2_-6,6	3.73; 3.81		63.52
Sucrose	CH-1 (Glc)	5.40	d	94.83
	CH-2	3.57		73.80
	CH-3	3.75		75.55
	CH-4	3.47		71.95
	CH-5	3.85		75.07
	CH_2_-6,6	3.80; 3.82		62.88
	CH_2_-1’ (Fru)	3.67		64.10
	CH-2′			104.2
	CH-3′	4.20		79.18
	CH-4′	4.04		76.93
	CH-5′	3.85		84.05
	CH_2_-6′,6′	3.83		65.73
α-fructose	CH_2_-1,1	3.58		65.70
	CH-3	4.09		78.18
	CH-4	4.09		78.18
	CH-5	3.82		83.36
	CH_2_-6,6	3,68; 3.77		65.48
β-fructose	CH-3	3.78		70.34
	CH-4	3.88		72.45
	CH-5	3.99		77.20
	CH_2_-6,6	3.69; 4.01		66.67
Organic Acids				
Lactic acid	α-CH	3.71	d	21
	β-CH_3_	1.22		
Aspartic acid	α-CH	4.00		
	β-CH_2_	2.71	dd	
	β′-CH_2_	2.84	dd	
Isobutyric acid	α-CH	1.08	d	36.2
	β-CH_3_	2.43	m	18.8
Acetic acid	CH_3_	1.94	s	
Formic acid	HCOOH	8.32	s	
Gallic acid	-CH	7.05	s	107
Other compounds				
Pinitol	H_3_	3.34	t	85.3
	-OCH_3_	3.59	s	
	H_4_	3.66	t	
	H_2_	3.81	m	
	H_1_ and H_6_	4.00	m	
Choline	−CH_3_	3.2	s	

In the ^1^H NMR spectrum (D_2_O, 300 MHz), H-1 at 3.880 (m,3H), H-2 at 3.676 (d, J = 9.9, 2.4), H-3 at 3.249 (d, J = 5.7), H-4 at 3.709 (d, J = 9.9),H-5 at 3.557 (m,1H, J = 9.6), H-6 at 3.624 (d,1H, J = 9.6) and the methoxy group as a singlet at 3.471.

### 3.2 Jellies preparation

**Fig. 4:**
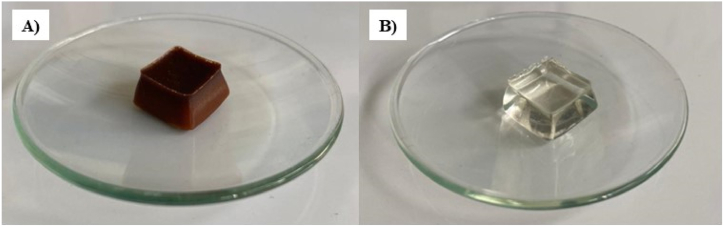
Jellies CGC based on carob flour (A) and CGS (B) acting as control.

The addition of CPF-based products to functional foods enhances antioxidant activity due to carob's natural compounds like polyphenols and flavonoids. Carob's organoleptic, nutritional, and biological properties make it a viable cocoa substitute, offering improved dietary fiber, and reduced sugars and calories. This substitution improves the nutritional value and technological and sensory properties of food products, making CPF an excellent candidate to replace cocoa powder in chocolate-like products, particularly for gluten-intolerant diets. CPF has been employed in the preparation of the jellies by mixing it with the commercial gelatin at 50 °C under magnetic stirring and pouring the semi-liquid mass into suitable molds allowing its refrigeration overnight at 4 °C (CGC) (Fig. 4A). The procedure was refined by adapting existing protocols from the literature, incorporating several modifications to enhance the final product's properties [18]. The proportions of gelatin to water and the gelatin/CPF weight ratio were meticulously selected to optimize the jellies' rheological, mechanical, and bioactive characteristics. These parameters are crucial as they directly influence the texture, elasticity, and health-promoting properties of the final product. Previous studies have shown that adjusting the gelatin concentration can significantly affect the gel strength and elasticity of gelatin-based products, while the addition of bioactive components like CPF can enhance the functional properties of the jellies [35,36]. By carefully controlling these ratios, the procedure aims to achieve a balance between texture and bioactivity, ensuring that the jellies are not only palatable but also beneficial to health. Similarly, to validate the physicochemical and biological properties of the preparation, a control sample was prepared by substituting CPF with commercial sucrose (CGS) (Fig. 4B). This allowed for a direct comparison between the two formulations, ensuring that any observed differences in rheological, mechanical, or bioactive characteristics could be attributed to the presence of CPF rather than other variables.

### 3.3 Antioxidant characterization of the carob flour and jellies

Carob pulp is a valuable source of polyphenolic molecules mainly gallic acid and its derivatives but can be also detectable minor components such as flavonoids, tannins, phenolic acids, and proanthocyanidins [37,38]. These chemical compounds contribute to the carob pulp's significant antioxidant, anti-inflammatory, and antimicrobial properties [39].

Quantifying phenolic molecules and characterizing their antioxidant features are essential to validate the health benefits of CPF-based jellies [40]. Each jelly underwent an extraction procedure at suitable time, with tests conducted after 0, 7, and 15 days. Table 3 presents the results for TPC, PAC, FC, and scavenging activity in both hydrophilic and lipophilic environments that clearly indicate as the substitution of sucrose with CPF significantly enhances the jelly's antioxidant performance. At day zero, TPC value for CGC was 19.31 mg of GA per gram of jelly, while CGS had a value one order of magnitude lower (1.71 mg of GA per gram). The value recorded for CGC has been in accordance with the amount of CPF in the preparation and slightly decrease (−6.1 %) over time, after a storage of 15 days. In PAC and FC assays, CGS showed no positive results, whereas CGC exhibited trends similar to TPC values. This one showed an 8.2 % decrease in phenolic acids after 15 days, while a more significant reduction (−18.6 %) in FC after 15 days has been recorded.

**Table 3:** Antioxidant properties of carob pulp flour (CPF) and jellies over a fifteen-day storage time (at 20 °C, in polyethylene bags).

Sample	Storage Time *(days)*	TPC *(mg GA g*^*−*^*^1^)*	PAC *(mg GA g*^*−*^*^1^)*	FC *(mg CT g*^*−*^*^1^)*	IC_50_*(mg mL*^*−*^*^1^)*	
					DPPH Radical	ABTS Radical
**CFP**		50.94 ± 1.31	10.89 ± 0.14	1.54 ± 0.06	0.082 ± 0.003	0.078 ± 0.003
**CGC**	1	19.31 ± 0.82^a^	4.26 ± 0.18^a^	0.59 ± 0.02^a^	0.191 ± 0.008^a^	0.158 ± 0.006^a^
	7	18.79 ± 0.54^a^	3.99 ± 0.15^a,b^	0.53 ± 0.02^b^	0.213 ± 0.009^b^	0.166 ± 0.006^a,b^
	15	18.13 ± 0.61^a^	3.91 ± 0.09^b^	0.48 ± 0.01^c^	0.235 ± 0.011^c^	0.175 ± 0.007^b^
**CGS**	1	1.71 ± 0.07^b^	–	–	–	–
	7	1.46 ± 0.05^c^	–	–	–	–
	15	0.99 ± 0.03^d^	–	–	–	–

CPF = Carob pulp fluor; CGC = Jellies based on carob pulp flour, CGS = Jellies based on sucrose; TPC: total phenolic content; PAC: phenolic acid content; FC: flavonoid content; GA: gallic acid; CT: catechin; DPPH: (2.2′-diphenyl-1-picrylhydrazyl); ABTS = (2.20-azinobis (3-ethylbenzothiazoline-6-sulphonic acid)). Each measurement was carried out in triplicate and data are expressed as means (±SD). Different letters express significant differences (p < 0.05).

CGC demonstrated strong scavenging capacities against the hydrophilic ABTS and lipophilic DPPH radicals at zero time. In an aqueous environment, CGC returned an IC_50_ of 0.158 mg mL^−1^, slightly increased (0.175 mg mL^−1^) after 15 days, consistent with previous tests. The same trend but reduced performance has been recorded in the organic environment against the DPPH radical. On the contrary, CGS showed no antioxidant activity anytime in each tested condition. These data highlight that the addition of CPF in the formulation not only increases the phenolic content, imparting significant antioxidant properties to the jelly, but also maintains these properties over time, despite a slight decrease.

Literature data confirm that carob from three different varieties contains the highest levels of biologically active compounds and represent a valuable source of polyphenol molecules [41]. Carob exhibited significant scavenging properties, as well as ferrous iron chelating capacity and ferric-reducing power. Additionally, existing studies indicate that flavonoid content varies significantly with harvest dates, peaking in the unripe stage and diminishing in the ripe stage [42].

Antioxidant features of the extracts displayed a direct correlation between TPC value and scavenger capacity, accounting for the contributions and potential synergistic effects of various antioxidant compounds in the sample. Given that different mechanisms can be involved in the biological antioxidant molecules performance, no single methodology can entirely estimate the antioxidant activity of a compound or food [43]. Therefore, the antioxidant profile of the extracts has been assessed by measuring their scavenger activity in both organic and aqueous media against DPPH and ABTS radical species, respectively. The recorded DPPH profiles yielded IC_50_ values consistent with literature data (0.0115–0.0304 mg mL^−1^) [44].

The antioxidant features of CPF have been effectively utilized in the formulation of jellies with significant antioxidant capacity against various radicals. The preparation of antioxidant jellies has been well-documented in the literature, achieved through the direct incorporation of natural extracts possessing notable biological properties. For instance, jellies formulated with red fruit puree and orange juice demonstrated antioxidant activity ranging from 50 to 83 mg of Trolox equivalent per 100 g [45]. Similarly, jellies containing berries and citrus extract exhibited antioxidant activity of 8.3–9.9 mg of Trolox equivalent per 100 g [46].

### 3.4 Rheological characterization

Rheological parameters provide valuable insights into the textural properties of materials. When a material returns to its original shape after external forces are eliminated, it exhibits elastic behavior. Conversely, if the material fails to return to its initial shape, it exhibits viscous or plastic performance [47,48]. In gelatin-based materials, the storage modulus (G′) reflects the gel's strength. Given the significant role of rheology in various applications, including gelatin gels, it is expected that rheological analysis will become an essential tool in future gel research.

**Fig. 5:**
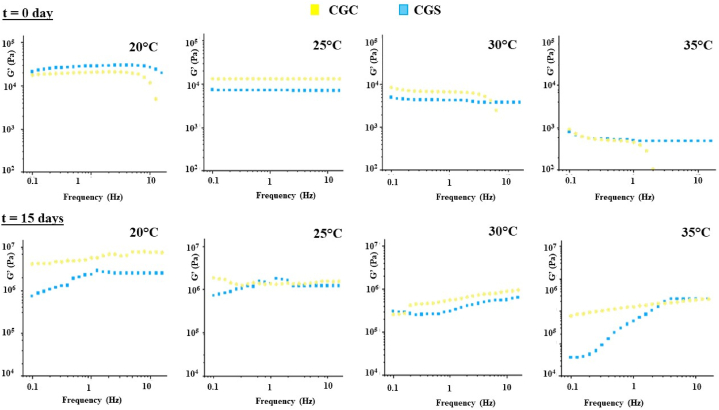
Frequency sweep tests at different temperature (20 °C, 25 °C, 30 °C, 35 °C) of CGC () and CGS ().

In Fig. 5, the elastic modulus (G′) profiles of all samples under different experimental conditions are presented, revealing several key rheological insights. Rheological measurements (Frequency sweep test) for both jellies indicate a near frequency independent G′ as is characteristic for gel materials [49,50]. The gel strength can be valued by the level of dynamic moduli [51]. The rheological spectra (Fig. 5) indicate the balance between the solid and viscous components of the jellies. Notably, G′ trend demonstrates the significant impact of CPF in the jellies formulation. Except for the sample at 20 °C, G′ is consistently higher in the CPF-enhanced jelly compared to the sucrose-based jelly, indicating improved consistency and elasticity [51]. Additionally, the carob flour maintains elasticity across varying temperatures. While the sample without CPF transforms into a weak gel at 35 °C, the sample with carob flour retains its strong gel characteristics even at higher temperatures, showcasing its superior thermal stability and reinforcing its role in enhancing jelly texture and elasticity [[52], [53], [54]].

### 3.5 Sensory test

**Fig. 6:**
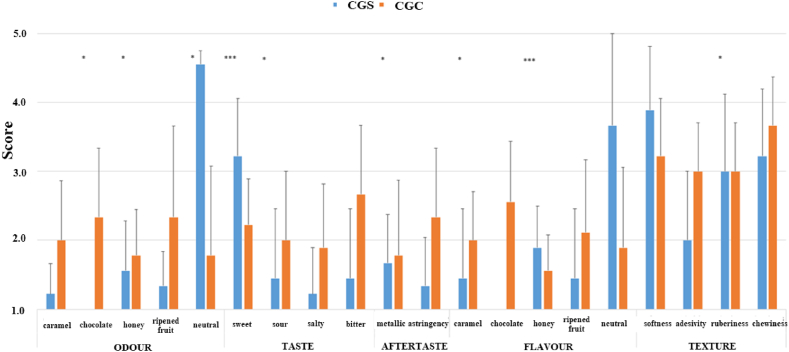
Sensory attributes (means ± standard deviations) evaluated by panelists. Asterisks indicate statistically significant differences for one way ANOVA test. ∗p < 0.05, and ∗∗∗p < 0.001.

Carob pulp flour added as ingredient in any food provides specific changes. As a matter of fact, the color is tremendously affected by the brown shades of this ingredient. Moreover, CPF imparts several qualitative attributes who may be perceived both as positive and negative [4]. Despite the growing CPF use as a cocoa replacer for many aromatic and chromatic similarities, CPF volatile profile is greatly characterized by fruity, nutty, and caramel notes, but also by beans, barley, and over ripened fruit whiffs [55]. In a recent work by Antoniou et al., 2023, the volatile profile of CPF was characterized; acids, esters, aldehydes, ketones, and alcohols were found in both grafted than non-grafted carob trees [56]. As known, the signature aroma of CPF is given by 2-methyl-propanoic acid resulting in cheesy and buttery notes. In a previous work [57] on a gluten free product, the inclusion of CPF gave nutty, rye, and gingerbread aroma to the final products, and some tasters referred to smell cheesy flavor. However, in the present paper, no attributes related to cheese were asked, for maintaining the focus on a sweet/confectionery product (jellies, candies) non containing milk and/or its derivatives. As visible from Fig. 6, panelists have recognized the neutrality of control samples during the tasting assigning always higher scores for CGS (4.6 ± 0.8, 3.7 ± 1.4 for odor and flavor classes respectively); among odors, chocolate impacted on the description of CGC jellies with a mean score of 2.3 ± 1.0 followed by ripened fruit 2.3 ± 1.8, and caramel 2.0 ± 0.9. Same attributes were statistically significant (p < 0.05) for CPF-based jellies in respect to control. Moreover, three panelists have found other descriptors defined as milk, coffee, and rye (score of 3 each), another panelist assigned the score 4 to the term other, without specifying. It is interesting to note how the presence of only sugar in control sample had anyway marked the perception of other attributes commonly related to candies and similar products. In particular, the term honey, both in odor and flavor cannot discriminate jellies. Moreover, the neutral flavor received quite a similar score of ripened fruit and honey. Several ketones and aldehydes were found in commercial samples of CPF and considered responsible of the waxy, pungent, nutty, woody, among others, volatiles [56].

CGC jellies had an increased bitterness, astringency, and a reduced sweetness in respect to the control sample, being statistically significative. These values are coming from the total elimination of sucrose from CGC jellies and the possible interaction of phenolic species mainly gallic acid and rutin known for their bitter taste [58].

For the texture, the inclusion of CPF has significantly (p < 0.05) increased adhesivity. In a recent study, it was found that CPF acts as a thickening and plasticizing ingredient thus mimicking sugar's role in candies [59]. Another study searched on the activity of CPF blended with other functional ingredients in cocoa/nuts spreads [60]. Even in this case, adhesivity was increased together with sourness and bitterness. Higher fiber content can combine oral saliva increasing the sensation of adhesivity and stickiness of CGC samples.

### 3.6 Antihyperglycemic and hypolipidemic effect

Spectroscopic characterization highlighted as CPF is an important source of several bioactive compounds such as D-pinitol and gallic acid. D-pinitol is a natural compound with potential antidiabetic effects, working through multiple mechanisms [[61], [62], [63]]. It acts as an insulin mimetic, enhancing glucose uptake by activating insulin signaling pathways. Additionally, it reduces insulin resistance by improving sensitivity and modulating insulin-responsive gene expression. Finally, it promotes glycogen synthesis and inhibits gluconeogenesis, reducing glucose production in the liver. D-pinitol as well as gallic acid also display remarkable antioxidant and anti-inflammatory properties, which decrease oxidative stress and inflammation, both contributing factors to diabetes. These combined effects help to improve glycemic control in diabetic individuals.

**Fig. 7:**
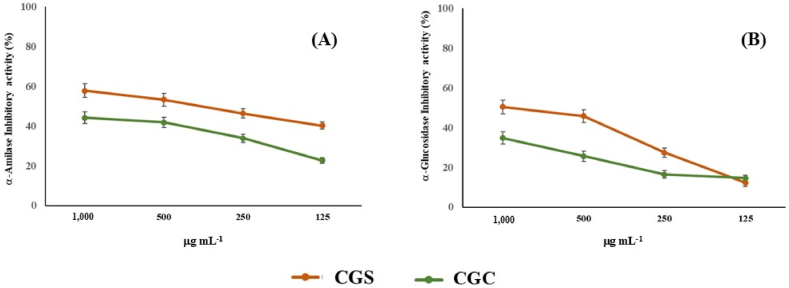
Carbohydrate hydrolyzing enzymes inhibitory activity of the jellies. (A) α-amylase; (B) α-glucosidase.

Generally, α-amylase enzyme resulted more sensible to the activity of carob. In fact, CPF-based jellies exhibited a dose-dependent inhibitory effect with a maximum percentage of inhibition of 57.7 % at maximum concentration tested (1.0 mg mL^−1^) (Fig. 7A).

A percentage of 50.3 % was recorded against α-glucosidase at 1.0 mg mL^−1^ for carob-based candy whereas control candy showed a percentage of 34.7 % at the same concentration (Fig. 7B). *Ceratonia siliqua* was traditionally used to the control the high blood glucose. Qasem et al. [64] demonstrated that carob exerted α-amylase and α-glucosidase inhibitory effect with IC_50_ of 93.0 and 97.1 μg mL^−1^, respectively. Furthermore, the *in vivo* effects of carob administration were evaluated in streptozotocin-induced diabetic rats, with doses of 500 mg kg^−1^ and 1000 mg kg^−1^ given to carob-treated diabetic groups. A dose-dependent hypoglycemic effect has been detected. In particular, rats treated with high-carob dose are characterized by a low glucose infusion test value. This hypoglycemic effect is probably related to the fibers in the carob responsible of the feeling of satiety [65]. At the same time carob jellies (CGC) are rich in polyphenols (Table 3). These phytochemicals such as polyphenols that could chelate different macromolecules such as lipids, fibers and carbohydrates, decreasing their intestinal absorption [66]. Recently, Ćorković et al. (2022) demonstrated that the hydroxylation of flavonoids, and the presence of caffeoyl moieties in phenolic acids improved the inhibitory activity against both carbohydrates hydrolyzing enzymes. Moreover, a possible synergistic effect among polyphenols and their metabolites was described [67]. More recently, Darwish et al. demonstrated the hypoglycemic effect of carob pod aqueous extract [68]. It is interesting to note that also carob stem bark and leaves are able to inhibit both carbohydrates hydrolyzing enzymes [69]. Despite the high carbohydrate content in carob, it demonstrated a significant antidiabetic effect, highlighting its potential as a valuable natural sweetener for individuals with hyperglycemic conditions.

**Fig. 8:**
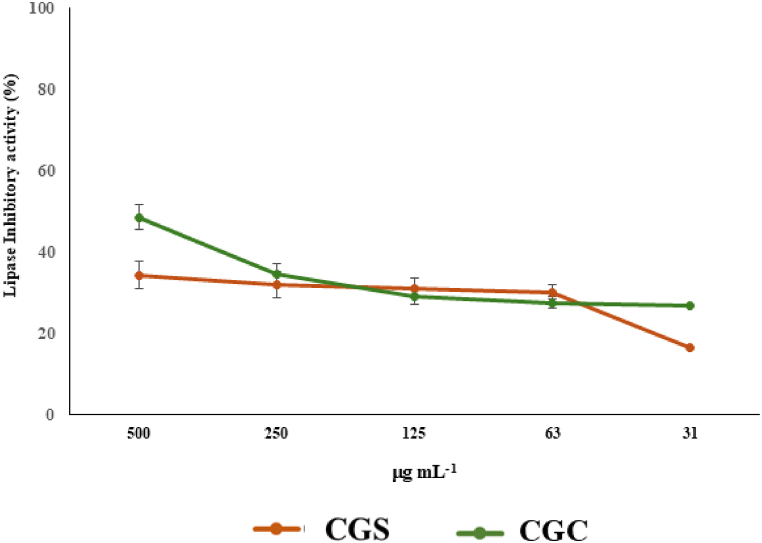
Lipase inhibitory activity of the jellies.

CPF-based jelly also showed a lipase inhibitory effect with percentage of 48.6 % at 5.0 mg mL^−1^ (Fig. 8). Previously, Jamos et al., demonstrated that both carob seed and leaves are able to exert a potent pancreatic lipase inhibitory effect with a percentage of inhibition of 95.4 and 84.0 % at 5.0 mg mL^−1^ [70]. Moreover, carob could prevent dyslipidemia *in vivo* through SIRT1/PGC-1α pathway as previously demonstrated [71].

## 4 Conclusions and future perspectives

This study examines the use of CPF as a sucrose substitute to produce high-value jellies with added health benefits. Spectroscopic analysis identified bioactive compounds in CPF, including D-pinitol, and gallic acid known for their beneficial effects on human health. Tests showed a high concentration of polyphenols and strong antioxidant activity. CPF-based jellies were successfully formulated and maintained their antioxidant properties over time, unlike sucrose-based jellies. Rheological analysis revealed that CPF jellies had better consistency, elasticity, and thermal stability, while sensory evaluations indicated that CPF jellies had increased bitterness and astringency, along with improved texture. Additionally, these jellies showed significant hypoglycemic effects, highlighting CPF's potential as a functional ingredient for healthier jelly products.

Despite CPF shows promise as a sucrose substitute in jellies, several areas need further exploration. The increased bitterness and reduced sweetness may impact consumer acceptance, highlighting the need for optimizing CPF concentration and flavor enhancement. Although CPF jellies retained antioxidant activity for 15 days, more research on long-term stability, texture, and shelf life is essential. Additionally, studies on the bioavailability and metabolic effects of CPF's bioactive compounds, as well as the economic feasibility and environmental sustainability of using CPF, are crucial. Expanding CPF's use in other food products and exploring additional health benefits could further unlock its potential.

In conclusion, while CPF shows significant promise in enhancing jelly products with health benefits, continued research into its sensory optimization, stability, bioavailability, and broader applications is vital for maximizing its potential and integrating it effectively into the food industry.

## Ethics declarations

The manuscript reports about a sensory analysis carried out thanks to the participation of voluntaries, trained and expert panelists belonging to the Department of Bioscience and Technology for Food, Agriculture and Environment of the University of Teramo, matching requisites for sensory tests as described in (ISO) 8589:2007. Considering the current regulations for ethics in food science research, we collected informed consent, while assuring data protection and privacy maintenance.

## Consent to publication

Not applicable.

## Data availability statement

The original contributions presented in this study are included in the article and its supplementary materials. No data were deposited in any publicly available repositories. Further inquiries can be directed to the corresponding authors.

## Funding

This research did not receive any specific funding.

## CRediT authorship contribution statement

**Umile Gianfranco Spizzirri:** Writing – review & editing, Writing – original draft, Validation, Methodology, Investigation, Formal analysis, Data curation, Conceptualization. **Luigi Esposito:** Writing – original draft, Formal analysis. **Paolino Caputo:** Writing – original draft, Formal analysis. **Maria Martuscelli:** Writing – review & editing, Writing – original draft, Supervision, Methodology, Investigation, Conceptualization. **Martina Gaglianò:** Formal analysis. **Maria Lisa Clodoveo:** Resources, Investigation. **Giuseppina De Luca:** Writing – original draft, Methodology, Investigation. **Cesare Oliverio Rossi:** Writing – review & editing, Writing – original draft, Methodology, Investigation. **Marco Savastano:** Writing – original draft. **Eva Scarcelli:** Formal analysis. **Monica Rosa Loizzo:** Writing – review & editing, Writing – original draft, Methodology, Investigation. **Donatella Restuccia:** Writing – original draft, Supervision, Methodology, Investigation. **Francesca Aiello:** Writing – original draft, Resources, Investigation.

## Declaration of competing interest

The authors declare the following financial interests/personal relationships which may be considered as potential competing interests:Donatella Restuccia reports a relationship with Cell Press that includes: board membership. Associate Editor-Food Nutrition Section (Heliyon) If there are other authors, they declare that they have no known competing financial interests or personal relationships that could have appeared to influence the work reported in this paper.
