# Supplementary material for: Carob pulp flour as an innovative source of bioactive molecules for the preparation of high-value-added jellies
Source: Heliyon. 2024 Sep 25;10(19):e38354. doi: 10.1016/j.heliyon.2024.e38354 (PMC11467575; doi:10.1016/j.heliyon.2024.e38354)
Supplement: Multimedia component 1 [file mmc1.docx]

**Supporting information section**

**Carob pulp flour as an innovative source of bioactive molecules for the preparation of high-value-added jellies**

Umile Gianfranco Spizzirri,^1,$^ Luigi Esposito,^2,$^ Paolino Caputo,^3^ Maria Martuscelli,^2,*^

Martina Gaglianò,^3^ Maria Lisa Clodoveo,^4^ Giuseppina De Luca,^3^ Cesare Oliverio Rossi,^3^

Marco Savastano,^5^ Eva Scarcelli,^6^ Monica Rosa Loizzo,^6^ Donatella Restuccia,^5,*^ Francesca Aiello^6^

^1^Ionian Department of Law, Economics and Environment, University of Bari Aldo Moro, 74123 Taranto, Italy; [g.spizzirri@unical.it](mailto:g.spizzirri@unical.it) (U.G.S.)

^2^Department of Bioscience and Technology for Food, Agriculture and Environment, University of Teramo, 64100 Teramo, Italy; [lesposito2@unite.it](mailto:lesposito2@unite.it) (L.E.); [mmartuscelli@unite.it](mailto:mmartuscelli@unite.it) (M.M.)

^3^Department of Chemistry and Chemical Technologies &UdR INSTM, University of Calabria, 87036 Rende, Italy;paolino.caputo@unical.it (P.C.); [martina.gagliano@unical.it](mailto:martina.gagliano@unical.it) (M.G,); [giuseppina.deluca@unical.it](mailto:giuseppina.deluca@unical.it) (G.D.L.); cesare.oliviero@unical.it (C.O.R.)

^4^Interdisciplinary Department of Medicine, University of Bari Aldo Moro, 70125 Bari, Italy;[marialisa.clodoveo@uniba.it](mailto:marialisa.clodoveo@uniba.it) (M.L.C.)

^5^Department of Management, Sapienza University of Rome, Via del Castro Laurenziano 9, 00161 Rome, Italy; marco.savastano@uniroma1.it (M.S.); [donatella.restuccia@uniroma1.it](mailto:donatella.restuccia@uniroma1.it) (D.R.)

^6^Department of Pharmacy, Health and Nutritional Sciences, University of Calabria, 87036 Rende, Italy; evascarcelli@outlook.it (E.S.); monica_rosa.loizzo@unical.it (M.R.L.); francesca.aiello@unical.it (F.A.)

***Correspondence:** [mmartuscelli@unite.it](mailto:mmartuscelli@unite.it); [donatella.restuccia@uniroma1.it](mailto:Donatella.restuccia@uniroma1.it)

**^$^**These authorsequally contributed to this work

**
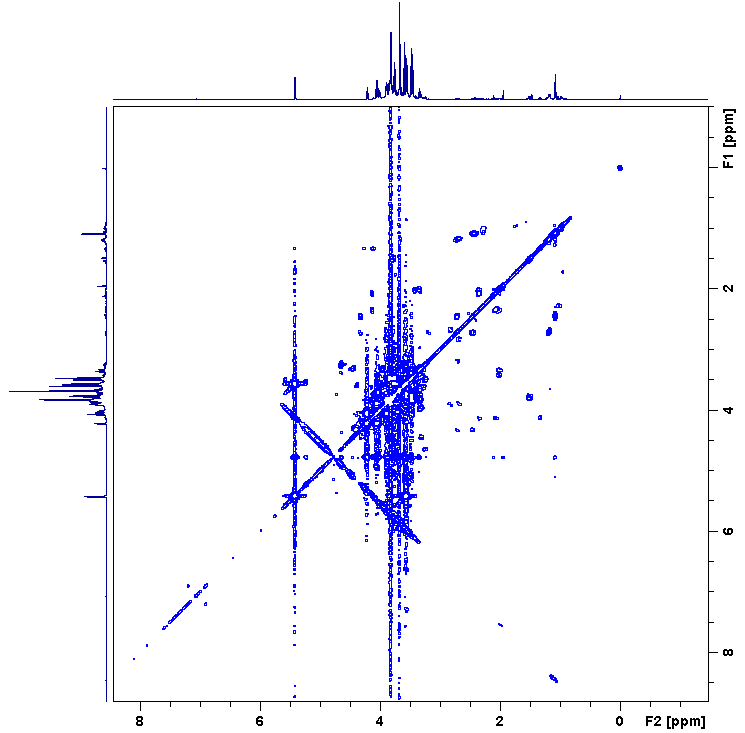
**

**Fig. S1.** 2D 1H COSY spectrum (Bruker pulse sequence: cosygpprqf) recorded on carob flour in D_2_O (field strength of 11.74 T).

**
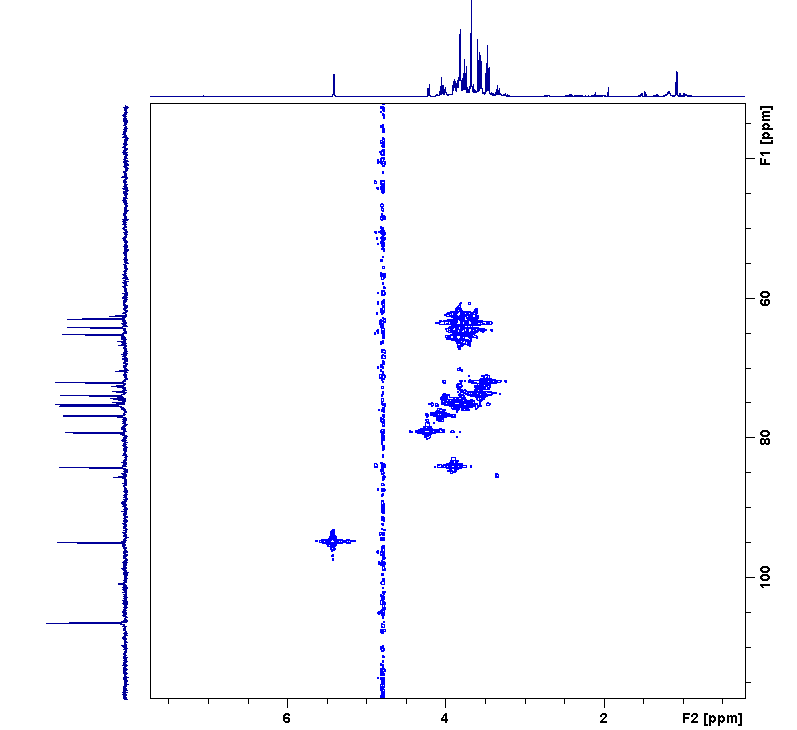
**

**Fig. S2.** 2D ^1^H-^13^C HMQC spectrum (Bruker pulse sequence:*hmqcgpqf*) recorded on carob flour in D_2_O (field strength of 11.74 T).
